# Blowing the whistle during the first wave of COVID‐19: A case study of Quebec nurses

**DOI:** 10.1111/jan.15365

**Published:** 2022-07-19

**Authors:** Marilou Gagnon, Amélie Perron, Caroline Dufour, Emily Marcogliese, Pierre Pariseau‐Legault, David Kenneth Wright, Patrick Martin, Franco A. Carnevale

**Affiliations:** ^1^ School of Nursing University of Victoria Victoria British Columbia Canada; ^2^ School of Nursing University of Ottawa Ottawa Ontario Canada; ^3^ Department of Nursing Université du Québec en Outaouais Gatineau Canada; ^4^ Palliative Care and Nursing Ethics, Centre for Research on Health and Nursing Ottawa Ontario Canada; ^5^ Faculty of Nursing, Université Laval Québec Canada; ^6^ Ingram School of Nursing McGill University Montreal Canada

**Keywords:** case study, COVID‐19, ethics, nurses, pandemic, qualitative, Quebec, whistleblower, whistleblowing

## Abstract

The experiences of nurses who blew the whistle during the COVID‐19 pandemic have exposed gaps and revealed an urgent need to revisit our understanding of whistleblowing.

**Aim:**

The aim was to develop a better understanding of whistleblowing during a pandemic by using the experiences and lessons learned of Quebec nurses who blew the whistle during the first wave of COVID‐19 as a case study. More specifically, to explore why and how nurses blew the whistle, what types of wrongdoing triggered their decision to do so and how context shaped the whistleblowing process as well as its consequences (including perceived consequences).

**Design:**

The study followed a single‐case study design with three embedded units of analysis.

**Methods:**

We used content analysis to analyse 83 news stories and 597 forms posted on a whistleblowing online platform. We also conducted 15 semi‐structured interviews with nurses and analysed this data using a thematic analysis approach. Finally, we triangulated the findings.

**Results:**

We identified five themes across the case study. (1) During the first wave of COVID‐19, Quebec nurses experienced a shifting sense of loyalty and relationship to workplace culture. (2) They witnessed exceedingly high numbers of intersecting wrongdoings amplified by mismanagement and long‐standing issues. (3) They reported a lack of trust and transparency; thus, a need for external whistleblowing. (4) They used whistleblowing to reclaim their rights (notably, the right to speak) and build collective solidarity. (5) Finally, they saw whistleblowing as an act of moral courage in the face of a system in crisis. Together, these themes elucidate why and how nurse whistleblowing is different in pandemic times.

**Conclusion:**

Our findings offer a more nuanced understanding of nurse whistleblowing and address important gaps in knowledge. They also highlight the need to rethink external whistleblowing, develop whistleblowing tools and advocate for whistleblowing protection.

**Impact:**

In many ways, the COVID‐19 pandemic has challenged our foundational understanding of whistleblowing and, as a result, it has limited the usefulness of existing literature on the topic for reasons that will be brought to light in this paper. We believe that studying the uniqueness of whistleblowing during a pandemic can address this gap by describing why and how health care workers blow the whistle during a pandemic and situating this experience within a broader social, political, organizational context.

## 1 INTRODUCTION

During the first wave of the COVID‐19 pandemic, defined as March to August 2020, Canada recorded 138,010 cases of COVID‐19 and close to 10,000 deaths (CPHA, 2021). The first wave had a devastating impact across the country, but not all jurisdictions were impacted equally (Flood et al., 2020). The province of Quebec, which is the second most populated province in the country and is home to approximately 22% of Canadians (Statistic Canada, 2021), was hit particularly hard during the first 5 months of the COVID‐19 pandemic. By the end of May 2020, for example, the province had recorded 45,773 COVID‐19 cases, accounting for 57% of all cases in the country (CPHA, 2021). By the end of July 2020, it had recorded close to 60,000 cases (INESSS, 2020). Of these cases, 14,191 (24%) were amongst health care workers (INESSS, 2020). It also recorded 5820 deaths, primarily in long‐term care (INESSS, 2020). Those deaths accounted for 65% of all COVID‐19‐related deaths in the country and were substantially higher than those recorded in other high‐income countries, including the United States (Urrutia et al., 2021). Because COVID‐19 disproportionately affected Quebec during the first wave, the province has been described as a ‘textbook case’ to study the COVID‐19 pandemic and government responses (Alami et al., 2021, p. 2). It also offers a real‐world case study of whistleblowing by health care workers during a pandemic and, more specifically, nurses working at the frontline.

## 2 BACKGROUND

The classic definition of whistleblowing is the one proposed by Near and Miceli (1985). The authors define whistleblowing as ‘a process involving at least four elements: (1) the whistleblower: a former or current member of an organization who is aware of wrongdoing but generally lacks the authority or power to make the required changes; (2) the whistleblowing act: the act of disclosing an illegal, immoral, illegitimate practice to persons or organizations that may be able to bring about change; (3) the complaint receiver: a third party (external whistleblowing) or someone other than or in addition to the immediate supervisor (internal whistleblowing); (4) the organization: a public or private organization who is the target of the whistleblowing and who will be called upon to respond (or not) to the disclosure of wrongdoing’ (Gagnon & Perron, 2020a, p. 381). Whistleblowing may appear to challenge to the authority structure of an organization, but it is not an act of deviance or a breach of loyalty per se; it is triggered by the seriousness of the wrongdoing and can indeed offer valuable information to improve organizational effectiveness and public safety (Gagnon & Perron, 2020a).

In the health sciences literature, much of the research focuses on the whistleblower (i.e. motivations, decision‐making processes, consequences of whistleblowing and so forth) and, to a lesser extent, on the context in which whistleblowing occurs and the process of whistleblowing itself (Gagnon & Perron, 2020a). The literature also takes as its starting point the experience of employees who witness wrongdoings in the workplace and disclose such wrongdoings internally or externally to the organization after careful ethical deliberation and weighing‐in of potential risks and consequences (Gagnon & Perron, 2020a). Nurses (and, to a lesser extent, nursing students) are the most studied health care workers in the whistleblowing literature (Mannion et al., 2018). We attribute this to the nature of nursing practice in care settings and nurses' extensive presence at the ‘bedside.’ Nurses also constitute the largest group of health care workers in the health care system, which increases their likelihood of witnessing serious risks or patterns of wrongdoings that may trigger a duty to act. Finally, they make up the frontline of the health care system, meaning that, in the event of a pandemic, they bear witness to the policy and management failures, the injustices and the toll these take on patients, families and other workers.

The nursing research on whistleblowing suggests that when nurses blow the whistle, they do so primarily out of concerns for patient care and outcomes (Jackson et al., 2014). Studies conducted to date have identified five types of situations that may result in whistleblowing: (1) unsafe working conditions, (2) deviations from practice standards; (3) unprofessional and harmful behaviours; (4) failure to uphold patients' rights and (5) management and organizational issues (Gagnon & Perron, 2020a). Nurses who sound the alarm in such situations are typically employees and they work within a particular organizational context that shapes their beliefs and values, decision‐making process, disclosure strategies and overall experience (Ahern & McDonald, 2002; Jackson et al., 2010a, 2010b, 2011, 2014; Mansbach & Bachner, 2010; McDonald & Ahern, 2000, 2002; Peters et al., 2011; Pohjanoksa et al., 2019ab). With regard to internal and external whistleblowing, recent studies by Pohjanoksa et al. (2019ab) suggest that whistleblowing trajectories are far more complex and messier than traditionally represented. One finding that is consistently noted across the nursing literature, however, is that nurses are more willing to blow the whistle internally (i.e. to follow the chain of command) (Pohjanoksaet al., 2019ab).

In many ways, the COVID‐19 pandemic has challenged foundational understandings of whistleblowing because it unfolded on a global scale, across technologically mediated societies, and at a time where health care workers are more connected than ever. Health care workers turned to online platforms, such as ProMED and Twitter, to sound the alarm in the early days of the pandemic (Lopreite et al., 2021; Wark, 2021) and leveraged social media tools to communicate to the public, warn decision‐makers, support each other and share testimonials as the pandemic was unfolding (Gagnon & Perron, 2020b; Glasdam et al., 2022). Health care workers also faced challenging working conditions compounded by a lack of personal protective equipment (Amon, 2020). They also witnessed and experienced the first‐hand impact of COVID‐19 policies, generating unprecedented moral distress and injury (Riedel et al., 2022). As a result, growing numbers of health care workers became whistleblowers and many faced reprisals for their actions (Amon, 2020). We believe that studying the uniqueness of whistleblowing during the COVID‐19 pandemic can address existing conceptual and empirical gaps by describing why (e.g. what types of wrongdoings, what motivations and to what ends) and how (e.g. nature of the process, steps followed, tools used) health care workers blow the whistle during a pandemic and situating this experience within a broader social, political, organizational context.

Quebec nurses constitute a novel case study to understand the experiences of nurses who blew the whistle during the COVID‐19 pandemic and identify key takeaways for decision‐makers, researchers, clinicians and nursing unions worldwide. The experience of Dr. Li Wenliang, the original COVID‐19 whistleblower who sounded the alarm on the Chinese messaging platform WeChat on 30 December 2019 and later died of COVID‐19 (Nie & Elliott, 2020; Zhu, 2020), serves as a strong reminder that whistleblowing in health care is not geographically bounded and that any effort to study the experience of whistleblowers during the pandemic is an opportunity to better support and protect health care workers. As such, the purpose of this paper is to present the findings of a case study that provides insights into the experiences of nurses who blew the whistle and offers potential avenues for improving supports to nurses moving forward.

## 3 THE STUDY

### 3.1 Aim

The aim was to develop a better understanding of whistleblowing during a pandemic by using the experiences and lessons learned of Quebec nurses who blew the whistle during the first wave of COVID‐19 as a case study. More specifically, to explore why and how nurses blew the whistle, what types of wrongdoing triggered their decision to do so and how context shaped the whistleblowing process as well as its consequences (including perceived consequences).

### 3.2 Design

**FIGURE 1:**
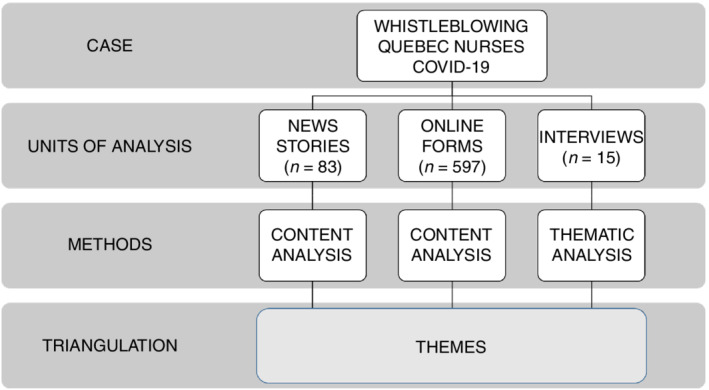
Case study.

We used a case study design as defined by Stake (2005) and Yin (2018) for two main reasons: Quebec's unique and dire context during the first wave of the pandemic and the exertive ways in which nurses engaged in acts of whistleblowing before and during COVID‐19. More specifically, we opted for a single‐case study design with three embedded units of analysis: news stories, online forms and semi‐structured interviews (see Figure 1). This design is appropriate when the selected case is unusual yet representative of a shared experience (i.e. blowing the whistle during a pandemic), and it has the potential to make a new and significant contribution to knowledge development (Stake, 2005; Yin, 2018). As such, the goal of single‐case studies is not to generalize from a single case but rather to conduct an in‐depth analysis of the selected case because it has the potential to reveal something new about a phenomenon (Stake, 2005; Yin, 2018). Consistent with single‐case studies, we completed the data collection and analysis for each embedded unit sequentially and then triangulated the three units to build a case description (Yin, 2018). We approached triangulation from an interpretive stance and included multiple data units to add ‘rigor, breadth, complexity, richness and depth’, not as a means of validation (Denzin, 2012, p. 82). In other words, we used triangulation ‘as an attempt to secure an in‐depth understanding of the phenomenon in question’ (Denzin, 2012, p. 82).

### 3.3 Data collection

Our single‐case study included three embedded units.

The first unit consisted of news stories published in Canadian media during the first wave of the pandemic. We completed our search using the Google Advanced Search operator, which provides the options of using and combining keywords, as well as limiting the search to a specific country (in this case, Canada) and specific dates (January–May 2020). We included the months of January and February because nurses were already blowing the whistle about pandemic preparedness and early response (before the first case of COVID‐19 was confirmed in Canada). We described our complete search strategy elsewhere (Gagnon & Perron, 2020b). After screening our initial sample of 119 news stories and eliminating duplicates, we included 83 news stories (Gagnon & Perron, 2020a, 2020b).

The second unit consisted of online forms completed by nurses and posted on ‘*Je dénonce*’ [I denounce], a public whistleblowing platform launched by the ‘*Fédération interprofessionnelle de la santé du Québec’* (FIQ) in March 2020. The FIQ is a union representing close to 80,000 health care workers in Quebec, the majority of whom are nurses. The platform was created to expose the experiences of frontline health workers, patients and families and to provide real‐time access to media, the public and decision‐makers. The online form allowed the user to report concerns about the pandemic mismanagement in care facilities and its impact on patients, families and staff. We retrieved 611 forms posted by nurses on the platform between March 2020, when the platform was launched, and May 2020. A total of 597 forms were deemed to meet our inclusion criteria: (1) submitted by nurses and (2) related to COVID‐19 (Perron et al., 2020a).

Finally, the third unit consisted of semi‐structured interviews conducted with Quebec nurses (September–December 2020). Nurses were recruited using e‐cards shared on social media and within existing professional networks. Participants were eligible to take part in this study if they: identified as a nurse (i.e. registered nurse, nurse practitioner or licensed practical nurse), practiced in Quebec during the first wave of the COVID‐19 pandemic and had a least one experience of whistleblowing during this period. Interviews were conducted in French or English, they lasted on average 60 min and were structured to cover four main domains. Each interview started by asking participants to describe their experience(s) of whistleblowing, including the type of wrongdoing(s), the people involved, the context and circumstances, the decision‐making process (including reasons motivating the decision and deliberation involved, if any), the whistleblowing process (including strategies used, reasons for using them and issues encountered) and the outcomes. Then, we asked about the organizational context, and more specifically about the organizational culture and how it shaped the whistleblowing process and the experience(s) more broadly. We also asked about the consequences of the whistleblowing, including professional and personal consequences. Finally, we asked participants to speak to lessons learned; in other words, what did the experience(s) teach them about themselves (as individuals and as nurses), about whistleblowing, about their workplace and about the broader health care system. We concluded by asking them if they had any advice to share with other nurses and if they wanted to offer recommendations for nursing organizations. All interviews were audio recorded and transcribed. Saturation was reached at 15 interviews.

### 3.4 Ethical considerations

News stories and online forms, both publicly accessible, did not require ethics approval. Approvals from the research ethics boards of the University of Victoria, University of Ottawa, McGill University, Université du Québec en Outaouais and Université Laval were obtained for the interview portion of the study. Informed consent was obtained prior to each interview. Pseudonyms were assigned to participants to ensure confidentiality. Compensation for the interview was provided.

### 3.5 Data analysis

Each unit of analysis was analysed separately using a ‘ground up’ approach (Yin, 2018) and sequentially, and then triangulated to generate the findings presented in this paper.

We analysed the news stories using a content analysis approach, which is particularly useful when working with large amounts of textual data (Hsiu‐Hsieh & Shannon, 2005; Schreier, 2014). We began by dissecting each news story using a series of questions: *Who is speaking? Are they speaking as a nursing collective (*e.g. *union, association, regulatory body) or as individual nurses? Are individual nurses anonymized or identified (*e.g. *full name; with or without picture)? What is being said in the news story? What is the essence of the message? How is the message changing over time?* Content extracted from this first round of high‐level analysis was then analysed inductively to identify common themes (Gagnon & Perron, 2020b).

We also used a content analysis approach to work through the online forms. However, given the size of the data set, we opted for a blend of inductive and deductive analysis. First, 50 randomly selected forms were analysed inductively to create a preliminary thematic structure. Then, 20 additional forms were analysed to ‘test’ our thematic structure, add emerging themes or combine existing themes. Using 10 additional forms, we confirmed there were no new themes and then worked through the rest of the data set by organizing it according to the thematic structure and frequency (Perron, et al., 2020a).

Interviews were analysed using Applied Thematic Analysis (ATA) (Guest et al., 2011). ATA involves four general steps: (1) read and code transcriptions, (2) identify possible themes, (3) compare and contrast themes, identifying structure amongst them and (4) produce a thematic scheme to describe the research phenomenon. We coded five interviews to identify broad themes that were then compared and contrasted as we analysed more interviews. These final organizing themes included: (1) Who is speaking (the whistleblower), (2) Where are they located (relationship to space and place), (3) What are they reporting (types of wrongdoings), (4) How are they reporting, using what strategies and why (whistleblowing process) and (5) What consequences of whistleblowing, real or perceived, do they describe (types of consequences).

Once the thematic analysis was completed, we moved to the final step in case study research: merging all three units to develop a case description (Yin, 2018). Case descriptions are particularly useful to descriptive case studies designed to offer new insights into a phenomenon and identify explanations that merit further exploration. Our case description sought to answer the following question: What can we learn from the experiences of Quebec nurses about the phenomenon of ‘whistleblowing during a pandemic’? To answer this question, we triangulated the three units to look for patterns and elements of explanations (Yin, 2018).

### 3.6 Rigour

We used a number of strategies to ensure rigour. Prior to conducting the study, we reviewed the literature and completed a concept analysis of whistleblowing in nursing (Gagnon & Perron, 2020a). We selected a case that meets the criteria for case study research and used multiple sources of data to gain a more in‐depth understanding the case (Yin, 2018). We maintained credibility by triangulating three units of analysis and maintaining prolonged engagement in the field (Houghton et al., 2013). We provided a rich description of the context to situate our findings and kept an audit trail of our process. Finally, we presented and discussed the findings with the aim of maximizing transferability while also pointing out unique elements of the case that other researchers can learn from and potentially use to inform future research.

## 4 SAMPLE

### 4.1 News stories

The voices of Quebec nurses were overrepresented in our sample (66%). Of the 83 news stories, 38 (46%) were published in English and 45 (54%) in French. News stories often included both collective and individual voices. Unions were the strongest collective voices in the sample, featured in 55% (*n* = 46) of the news stories. The FIQ was by far the most active union voice, appearing in more than half of those stories. We found equal numbers of stories in which nurses were identified (with name and picture) and anonymized. The main reason cited for requesting anonymity was the risk of workplace retaliation and sanctions (including job loss) for speaking out in the media.

### 4.2 Online forms

Our sample exclusively included online forms submitted by nurses. The online form included the option of entering personal information (i.e. name, title, workplace). However, in the majority of our sample, nurses opted not to disclose such information. The form also included a text box and the option of attaching a document or a picture. Our sample included the text box content of the forms submitted by 597 nurses.

### 4.3 Interviews

We interviewed 15 nurses, including licensed practical nurses (*n* = 1) and registered nurses with a college (*n* = 4) or a university (*n* = 10) degree. The majority of participants reported practising in a hospital setting (*n* = 9), while others reported practising in long‐term care (*n* = 4) and home care (*n* = 2). Out of the 15 participants, 14 self‐identified as women. Most participants were 35 years old or less (*n* = 9). Twelve (80%) participants had been working as a nurse for 10 years or less (6 with 5 years or less of experience, 6 with 5–10 years of experience). Finally, when asked about previous experiences of whistleblowing, half of the participants reported having blown the whistle at least once before COVID‐19.

## 5 FINDINGS: THE CASE

In accordance with case study methodology, our findings will be divided into two sections. The first section details contextual conditions before and during COVID‐19. Such conditions are paramount to understanding the case and interpreting the themes identified in the data. The second section presents the themes identified across all three units of analysis.

### 5.1 Contextual conditions

#### 5.1.1 Before the COVID‐19 pandemic

Already before the pandemic, acts of whistleblowing by Quebec nurses regularly made headlines due to several factors. Interestingly, these same factors fueled the devastating effects/experience of the first wave in Quebec and forced nurses to scale up their external whistleblowing strategies. One important factor, as stated by Alami et al. (2021), was the 2015 reform of the Quebec health care system, which merged 182 health and social service organizations into 34 megastructures of 12,000–15,000 employees, followed by extensive financial and staffing cuts to management and public health. This reform profoundly impaired, and in some instances eradicated, internal communication channels normally used by nurses to report wrongdoing to management. Another factor was the persistent and normalized use, for nearly two decades, of mandatory overtime as a routine management strategy to address the nursing shortage, despite extensive evidence that it discouraged full‐time employment, fueled nurse burnout and departure, and hindered retention efforts (Quebec Nurses Association, 2019). In other words, by normalizing the use of emergency powers as a routine management strategy, Quebec entered the pandemic with a weakened nursing workforce whose capacity to handle increased demands, high patient loads and forced overtime was exceedingly jeopardized.

The culture of silence across the health care system was an important factor denounced before the pandemic. This culture favoured the muzzling and disciplining of whistleblowers to the detriment of transparency and organizational change. It is worth recalling that in 2017, the Quebec government implemented the *Act to facilitate the disclosure of wrongdoings relating to public bodies*. The main purpose of this law was to protect whistleblowers in the public sector, including in publicly funded health care settings and facilities. However, instead of facilitating whistleblowing, it has been found to provide a narrow pathway for disclosure and impose conditions under which public disclosure can be made—and protection granted. In view of the above, Quebec entered the first wave of the COVID‐19 pandemic with a law that did not significantly change the culture of silence in health care, nor did it offer concrete disclosure tools or mechanisms for nurses to use.

#### 5.1.2 During the COVID‐19 pandemic

In addition to the three pre‐existing factors described above, it is important to highlight a number of pandemic‐related events and decisions that further fueled nurse whistleblowing in Quebec. On March 21, 2020, the Minister of Health and Social Services issued a Ministerial Order (2020‐007) under the *Public Health Act* to suspend multiple clauses of health care workers' collective agreements and allow employers to cancel union leaves, suspend, cancel or defer vacation time, redeploy staff, change work schedules, force employees to work full‐time and suspend mechanisms for grievances and arbitrations. As we write this paper, this Ministerial Order remains in effect. On March 30, 2020, the FIQ launched its public whistleblowing platform ‘*Je dénonce*’ [I denunce], which we described above. That same week, the Quebec government announced a new online portal called ‘*Je contribue!*’ [I contribute!] to recruit working or retired members of the public as volunteers to support the government's pandemic response, particularly in long‐term care. A month later, the Canadian government announced it was sending the Canadian Armed Forces (CAF) to help in Quebec's long‐term care facilities.

On May 16, 2020, in light of the overwhelming success of ‘*Je dénonce*’ [I denunce] and the resulting exposure of pandemic mismanagement across care settings, the Ministry of Health and Social Services announced the creation of a new email box called ‘*On vous écoute*’ [We are listening] to encourage health care workers to report issues directly to the Ministry. This initiative, an attempt to ‘end the culture of silence’ as stated by the Minister of Health and Social Services at the time (Fréchette & Béfort‐Doucet, 2021, p. 18), was met with criticisms for several reasons: It failed to address the long‐standing culture of silence in the health care system; it lacked transparency; it did not provide safeguards for health care workers who reported concerns; it redirected nurses away from external whistleblowing channels (e.g. social media, media and ‘*Je dénonce*’ [I denunce]) towards internal ones and, crucially, it removed critical pandemic management problems from public view, thus eliminating a key tool for transparency and accountability. Later that month, the CAF released a damning report documenting critical failures in long‐term care facilities, where the highest COVID mortality rate occurred (a public inquest into long‐term care deaths has just concluded). During the first wave, close to 4000 long‐term care residents died of COVID‐19 in Quebec. More than 15,000 cases were recorded amongst residents and staff, which represents 60% of all long‐term care cases in the country (CIHI, 2021). At the peak of the first wave, Quebec was ‘reportedly the seventh deadliest place in the world’ (Flood et al., 2020, p. 6).

### 5.2 Key themes within the case

**FIGURE 2:**
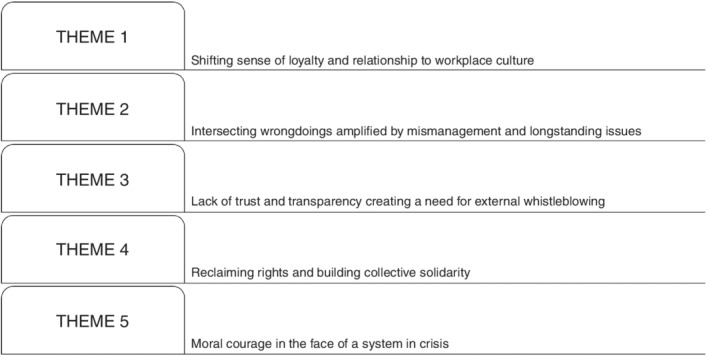
Themes.

We identified five themes across our case (see Figure 2). Together, these themes elucidate why and how nurse whistleblowing is different in pandemic times. While these themes are specific to the case (i.e. whistleblowing amongst Quebec nurses) and the contextual conditions outlined above, they offer a more nuanced understanding of nurse whistleblowing and address important gaps in knowledge. As previously mentioned, whistleblowing research and theory have traditionally taken as a starting point the experience of employees who witness particular wrongdoing in the workplace and disclose such wrongdoings—usually internally first, then externally when internal strategies have failed (Gagnon & Perron, 2020a). During the first wave of the pandemic, however, nurses across all units of analysis repeatedly witnessed exceedingly high numbers of intersecting wrongdoings that entailed significant risks regarding the rights, safety, health and well‐being of both patients and staff. This resulted in high levels of moral distress. In nurses' view, the urgency of the situations (e.g. high or immediate risks)—and the crisis context more broadly—justified swift actions and led to a shift in their perceptions of whistleblowing, wherein anticipated benefits of disclosing wrongdoings outweighed other considerations. Nurses' sense of loyalty and obligation was also in flux for the reasons outlined below. Finally, they were privy to information about the risks and the harms associated with the pandemic response that, for the most part, was unknown to the public—and to some extent, to elected officials and policymakers.

#### 5.2.1 Shifting sense of loyalty and relationship to workplace culture

Our findings revealed that Quebec nurses who blew the whistle during the first wave of COVID‐19 did not experience the classic ‘clash of loyalty’ (employer vs patient) widely described in the whistleblowing literature. It was evident across our sample that COVID‐19 measures and the overall response had contributed to a shift in loyalty for nurses who felt discredited, ignored, instrumentalized, devalued and abandoned by employers and the government.

I knocked on all the doors. I wrote a 16‐page testimonial asking management for help. I sent emails. I wrote on Messenger, I called … But no one came, no one came to help. (Online form 840)

The many workplace safety issues faced by nurses, especially the lack of access to adequate personal protective equipment (PPE), contributed to this shift in loyalty. Our sample included hundreds of testimonials from nurses reporting insufficient PPE as well as situations in which employers knowingly and intentionally rationed, locked away and denied access to PPE. Nurses felt that employers breached their social contract and jeopardized patients' and employees' safety, health and lives. In fact, one of the most common feelings expressed in our data was that of feeling ‘disposable.’

We feel like objects, like pawns that are being moved on a chessboard. We are being held hostage. “This ministerial order is a bomb,” she says, “Mr. Legault (the Premier),” you are losing your guardian angels. We are not numbers, we are not robots. (Anonymous nurse quoted in a news story published on April 30, 2020)

Interestingly, nurses who engaged in public (external) acts of whistleblowing, often fully identified, had a different relationship to workplace culture. Nurses who volunteered in long‐term care facilities, for example, were amongst the most vocal of all nurse whistleblowers. We found that having no ties to these facilities, no contractual agreements and no perceived duty of loyalty to management significantly influenced how nurses blew the whistle. In other words, being an ‘outsider’ facilitated external whistleblowing. Furthermore, for nurse volunteers, their status allowed them to resign easily from their position and escalate their whistleblowing efforts:

I think it [whistleblowing and resigning] was easier for me, being a volunteer and not attached to the long‐term care home. My job was not on the line. The long‐term care home was not my employer. (Blandine, October 22, 2020)

The high‐profile testimonial of nurse Nadia Lambert, which was initially posted on Facebook, shared 20,000 times in 24 h and relayed across multiple news outlets, illustrates how having no relationship with care settings and, most importantly, nothing to lose played a role in shaping nurses' whistleblowing strategies. Lambert volunteered 8 days in long‐term care before resigning, raising the alarm on appalling care conditions that were later documented in the CAF report: extreme understaffing, lack of PPE and equipment (e.g. thermometer, saturometer, etc.), abandonment of residents in unsafe and unhygienic conditions, residents experiencing hunger and dehydration, unsafe transfers leading to preventable COVID‐19 transmission and deaths.

#### 5.2.2 Intersecting wrongdoings amplified by mismanagement and long‐standing issues

Our findings depart from the traditional definition of whistleblowing as a process that seeks to expose one particular type of wrongdoing (e.g. wrongful practice, illicit behaviour, criminal conduct). During the first wave of COVID‐19, nurses faced a high number of intersecting wrongdoings on a daily basis related to insufficient PPE and staffing, unsafe working conditions, excessive mandatory overtime, communication breakdowns and breaches of infection prevention and control protocols. These wrongdoings manifested in practice, but they were managerial and political in nature: they arose from the mismanagement of the pandemic and government actions, most notably the issuing of the Ministerial Order in March 2020. This order suspended nurses' collective agreement, curtailed their labour rights and degraded their working conditions, creating a fertile ground for increased risks and unchecked abuses by management:

I find it strange that my organization is using the Ministerial Order. They are already cancelling vacations and holidays, and imposing mandatory overtime, but there is no need for it. I work in the Intensive Care Unit, and there are no COVID‐19 cases because we are transferring them automatically. We are not eligible for the COVID‐19 bonus for that reason! Why use the Ministerial Order when we don't have a bonus for the exact reason that we are not experiencing a COVID‐19 crisis? We have a staff surplus every day, but they are forcing nurses to work full‐time regardless? My organization is using powers granted by the government even though it doesn't need to, just because they can! (Online form 797)

Across our sample, nurses described the Ministerial Order as a blunt instrument that was overused (and abused) in the health care system. In particular, nurses denounced the fact that the order exacerbated many long‐standing issues (e.g. nurse burnout) entrenched by a 20‐year legacy of using exceptional measures such as mandatory overtime as a routine management practice rather than addressing deteriorating care environments. As one interview participant explained, Quebec nurses were already frequently working 16‐h shifts in mandatory overtime before COVID‐19. As one participant noted, following the Ministerial Order:

The imposition of mandatory overtime increased a lot. The increase was phenomenal (…) For example, there was one shift where the unit was five nurses short, and they didn't find anyone. All the nurses were forced into overtime, and they all did a 16‐h shift. (Hélène, November 11, 2020)

During the interview, Hélène further described an instance of being forced to work 24 consecutive hours and waking up the next day feeling ‘hungover’ from severe fatigue. That day, she decided to undertake several steps to blow the whistle. She explained her breaking point:

I had a major panic attack the next day. This experience really shook my mental health. I was questioning myself. At first, I was wondering if I was the problem or if I was unable to give enough at work. I thought that maybe I was deficient as a nurse, you know? Working 16 h is so normalized. Same for working crazy shifts while putting yourself second and having no regard for your needs, desires, and passions. And finally, I came to the conclusion that the problem wasn't me, that I wasn't sick. The health care system is sick. (Hélène, November 11, 2020)

The Ministerial Order also exacerbated another long‐standing issue in the Quebec health care system: the culture of silence. As employers enacted policies preventing nurses from speaking to the media or posting on social media and threatened nurses with sanctions (e.g. discipline, suspension, dismissal), nursing unions, online nursing support groups and individual nurses became more vocal. Whistleblowing became imperative not only because of multiplying wrongdoings experienced in the workplace but also because of mounting pressures to muzzle nurses. As FIQ then‐President Nancy Bédard explained in one news story:

Nurses are exasperated. They are abused right now. We give them minimal PPE, we don't protect them. They are exhausted. They are hearing that their right to paid vacation will be limited. We are forcing them to work two weekends out of three. Their schedules are changed because of the Ministerial Order. The Minister issues orders and grants powers to health care organizations that then, in turn, create working conditions that are extremely difficult and baffle their rights (…). What we want is for nurses to be able to speak freely. (May 16, 2020)

#### 5.2.3 Lack of trust and transparency creating a need for external whistleblowing

Nurses engaged in internal whistleblowing or external whistleblowing, or both. Internally, the two main strategies mentioned across the sample were emailing one's superior or using institutional forms such as incident reports. Nurses shared that these strategies were ineffective, and for many, they even resulted in targeted retaliation and sanctions, including reprimand letters and suspensions. Notably, many nurses who wished to sound the alarm internally were unable to find effective means to do so within the organizations where they worked or volunteered. One interview participant, who made multiple attempts to report serious issues internally, summarized it as such:

Basically, I realized that not only did official [internal] channels not work but there aren't any, really, like they don't really exist. I asked everyone, “What is the official channel for reporting this [lack of PPE]?” and no one knew. They all said “maybe this or that,” but no one was able to tell me (…). It was eye‐opening for me. I knew that official channels were like an illusion, that they didn't really work, but then I experienced it. Trying to find them, and they just don't exist. (Gabriella, October 16, 2020)

Data across all three units showed that institutional reporting channels were already deficient or inexistent before the COVID‐19 pandemic. Furthermore, for many nurses, the decision to turn to outside channels was also due to the fact that the issues they needed to report were organizational, making internal reporting channels less safe. There was a clear lack of trust in organizations and, more broadly, in the Ministry of Health and Social Services. Most importantly, nurses felt that reporting internally would fail to generate the needed response to address critical issues in a timely manner or, worse, would further silence nurses and keep these issues hidden from public view. As a result, many nurses privileged external whistleblowing strategies, including social media posts on individual and group pages, social media campaigns, media interviews and public testimonials through FIQ's whistleblowing platform ‘*Je dénonce*’ [I denunce]. We found many examples of one of these strategies leading to another. For example, media accessing and citing public testimonials posted on ‘*Je dénonce*’ [I denunce] as they reported on the pandemic:

We are admitting patients diagnosed with pneumonia. No COVID‐19 testing for these patients. No access to N‐95 masks. The staff working on units with suspected COVID‐19 patients are sent to work on other units the next day. (Anonymous nurse testimonial posted on ‘*Je dénonce*’ [I denunce] and featured in a news story published on March 31, 2020)

External whistleblowing strategies were motivated by the seriousness and urgency of the issues faced by nurses as well as the need for greater transparency. By acting as a conduit between nurses, media outlets, decision‐makers and the public, the FIQ platform filled an important informational gap and mitigated the risk of blowing the whistle externally. It also provided a way for nurses to report unfair labour practices for which they could no longer file a grievance under the Ministerial Order. Thus, we found strong evidence of nurses using the platform to report labour rights issues such as this one:

My superiors are aware of my immunocompromised status, but they are refusing the medical note written by my specialist. They told me I will be assigned to the COVID unit Sunday. (Online form 824)

Reflecting a lack of trust and a need for transparency, nurses were critical of the Ministry's ‘*On vous écoute*’ [We are listening] email box, citing the lack of anonymity, the opacity of the process, the absence of public accountability mechanisms and its stated aim of deterring nurses from blowing the whistle publicly.

#### 5.2.4 Reclaiming rights and building collective solidarity

We found that whistleblowing, in particular external whistleblowing, was not only used to publicly expose wrongdoings and advocate for a better pandemic response, but also to reclaim rights that were thwarted through the Ministerial Order and by the ramping up of managerial efforts to silence nurses. Our analysis of the textual data and interviews shows that speaking up and speaking out during the first wave of COVID‐19 became imperative. While media reporters extensively described nurses' testimonials as a ‘cry for help,’ we argue that they reflected instead nurses' reclaiming their right to speak. One interview participant who did media interviews explained:

It [speaking to media] lifted a weight off my shoulders. To say out loud what others [nurses] were thinking to themselves and did not dare to say (…) I thought to myself that a door was open for me and it wasn't for nothing, so I did it [speak to media]. When I did it, I realized that I didn't care if I got retaliations…Not sure what they would be really, but I just thought to myself that we're in a free country, and we have the right to speak. (Josette, 5 November 2020)

External whistleblowing also allowed nurses to speak as *workers* and push back against the predominant ‘angel narrative.’ Indeed, during the first two waves, the Quebec Premier consistently described nurses as guardian angels during daily briefings, which nurses viewed as a perverse strategy to encourage and normalize nurses' sacrifice. External whistleblowing allowed the production and dissemination of a counter‐narrative re‐centering nurses as human beings and as workers entitled to protection from unsafe working conditions.

They call us guardian angels, but they treat us like numbers. I'm immunocompromised and failing my fourth treatment for multiple sclerosis. They are refusing my neurologist's medical note and denying my leave. I'm a guardian angel, so I have to continue working despite his advice. I'm a nurse, but I'm seen as a guardian angel. I'm not treated as one. For years now, we've been overworked with more complex patients. We give everything at work, to the detriment of our families (…) In short, Premier Legault, stop calling us guardian angels (…). (Online form 9)

Nurses' desire to be seen and treated as human beings was one of the most consistent threads across the sample. It was also a powerful driver of external whistleblowing because it provided an opportunity to represent nurses as health care workers whose inherent dignity and vulnerability to COVID‐19 demanded recognition, as opposed to disposable caregivers expected to sacrifice themselves. We found that efforts to humanize nurses were part of a broader struggle for nurses to reclaim their rights as workers and speak out against governmental and managerial decisions that put their health, lives and families at risk. These included, but were not limited to, being denied COVID‐19 testing, being forced to work while symptomatic, being refused workplace accommodations, being placed in high‐risk situations (i.e. pregnant nurses, immunocompromised nurses, etc.) and having insufficient/inadequate or no access to adequate PPE. One national media outlet reported a story about a nurse who was denied testing as follows:

That he was told after his shift that he had been exposed to a nurse who tested positive for COVID‐19. When he requested testing, “he was denied testing by the hospital.” At the time of the interview, he [the nurse] insisted on the importance of “testing every single nurse out there.” When asked to comment on the news story, the Quebec Health Minister responded that “testing is a priority,” but residents and patients come first. (Quoted from a video interview, Global News April 16, 2020)

Throughout the sample, we noted that external whistleblowing strategies used by nurses had a strong collective focus; that is, when nurses spoke out and spoke up about their individual experiences, they did so in solidarity with other nurses and for their collective rights. As mentioned above, we also found a high number of news stories featuring collective union voices. This is an important finding because whistleblowing tends to be understood exclusively as an individual phenomenon.

#### 5.2.5 Moral courage in the face of a system in crisis

Our findings suggest that blowing the whistle was experienced and seen as an act of moral courage by nurses. Although slight variations exist in the definition of moral courage, we define it here as the courage a person demonstrates when acting in a way that aligns with their values and beliefs despite fear or threat of negative consequences for the acting individual (Pajakoski et al., 2021). We use the concept of moral courage to capture the motivations, rationales and intentions cited by nurses across all units. Nurses' decisions and actions were first and foremost motivated by a strong sense of moral and professional obligation to advocate for patients. One nurse who resigned from long‐term care after witnessing the deaths of many residents due to COVID‐19 spoke to the media after writing a letter to the Premier, the Minister of Health and the Director of Public Health. She explained what motivated this decision and action:

We've been screaming for help for a long time. This crisis [the COVID‐19 pandemic] exposed the existing flaws in our health care system and how extensive they are. Yes, we [nurses] want to be there and help, but our role as nurses is also to be advocates for our patients. (nurse quoted in a news story published on April 25, 2020)

The rationales underpinning nurses' decisions and actions can be divided into three main categories. The first category focuses on the *wrongness* of the pandemic response and how it created and exacerbated COVID‐19‐related risks, suffering and deaths. The second category speaks to the need to do *the right thing*. Nurses were adamant that blowing the whistle, through whatever means necessary, was the right thing to do as nurses because it was in the public's interest, consistent with professional obligations, and a matter of moral integrity. The third category, which emerged clearly and strongly in our data, was the realization on the part of many nurses that they had nothing (or less) to lose and nothing (or less) to fear anymore. Nurses felt that in a system in crisis that desperately needed nurses, they held more power, and they, therefore, assessed the risks of whistleblowing differently than before COVID‐19. While most perceived fewer risks, leading them to act without or despite the fear of negative consequences, for some such fear remained and was the main reason for requesting anonymity in media interviews or for resorting to other reporting strategies, such as the FIQ platform, for example.

We're at a point of wanting to quit collectively. Our employer tried to intimidate us recently at a meeting. One of the nurses got a disciplinary notice yesterday, and she quit on the spot, so the day staff had to do mandatory overtime. (Online form 571)

In addition to the motivations and rationales described above, nurses had clear intentions when they blew the whistle. They were hoping for change, but they were also determined to bring much‐needed awareness to the public, the media and the government about pandemic management failures. In order words, they strove to make the invisible visible. This explains why many of our interview participants stated that blowing the whistle gave them a feeling of ‘mission accomplished,’ regardless of th outcomes.

I guess the moral distress of being too scared to even say anything about it, so you're going to feel worse in the long term. I think that's something that's been really true for me. After I blew the whistle—in both counts [of whistleblowing]—I felt like I fulfilled my duty, like I…There was something wrong, I spoke out about it. And that's all I can do as myself. So I've fulfilled my moral obligation, in that sense. (Anya, October 23, 2020)

## 6 DISCUSSION

This paper adds to a growing body of evidence on the experience of health care workers, especially nurses, during the COVID‐19 pandemic. Whistleblowing has been and continues to be central to that experience. The case study of Quebec nurses during the first wave is a helpful empirical approach to understanding why and how whistleblowing during a pandemic differs from whistleblowing in other (non‐crisis) contexts. Our findings highlight five main differences, which are likely transferable to other jurisdictions and future pandemics (or public health crises). First, nurses did not perceive a ‘clash of loyalty’ as is typically described in the literature (Gagnon & Perron, 2020a). This was true for nurses who blew the whistle both as employees and as volunteers. They felt a strong sense of loyalty to the profession, patients and the public, but given widespread managerial abuses and the risks they faced, they did not believe they owed loyalty to employers, institutions and the government. Second, the nature of the wrongdoings witnessed by nurses was managerial and political in nature. This departs from existing literature, which mainly locates wrongdoings within a specific person (e.g. colleague, manager, etc.), workplace or institution. Rarely does the literature on whistleblowing in health care speak to system‐level wrongdoings. Third, nurses did not follow the traditional whistleblowing pathway, which typically begins with the nurse using internal reporting channels before resorting to external whistleblowing when they lose trust in internal channels (e.g. following retaliation) and/or determine that these channels are ineffective. Instead, they turned to external whistleblowing far more quickly, hoping for a prompt, more efficient remediation. Furthermore, nurses across our sample used technologically mediated external whistleblowing strategies that have not been widely studied in nursing. Fourth, external whistleblowing served to reclaim nurses' right to speak and build solidarity amongst nurses. Again, this is not typically documented in the literature, given researchers' emphasis on the whistleblower as an individual and the act of whistleblowing as solely an individual undertaking. Finally, blowing the whistle during a pandemic emerged as an act of moral courage hinging on a shifting understanding of risk, duty and power. This has not been documented in the nursing literature to date.

### 6.1 Rethinking external whistleblowing

Nursing has a complicated relationship with external whistleblowing. At the level of the profession *and* the discipline, external whistleblowing is typically depicted as a last resort, a risky practice and an act of disclosure that may run counter to professional and contractual duties—thus leaving nurses with little protection and support (Gagnon & Perron, 2020a). This approach to external whistleblowing not only shapes the experiences of nurses who blow the whistle but it also governs how we study and think about those experiences. As a result, the nursing literature tends to focus on the whistleblower's beliefs and values (Ahern & McDonald, 2002), decision‐making process (Jackson et al., 2010a; Pohjanoksa et al., 2019a, b) and consequences (Jackson et al., 2010b, 2011; McDonald & Ahern, 2000, 2002; Peters et al., 2011). In other words, research to date focuses on how nurses come to make the ‘difficult’ decision to blow the whistle, which is assumed to only be ethically justifiable in exceptional circumstances and inherently risky, and on the consequences they may face as a result. Less emphasis has been placed on organizational culture and its role in increasing or reducing the need for external whistleblowing, harming or supporting nurse whistleblowers, problematizing or normalizing disclosures of wrongdoings and so forth (Gagnon & Perron, 2020a; Jackson et al., 2014). Our findings suggest that external whistleblowing is a symptom of a system in crisis, one that triggers an obligation on the part of nurses to speak courageously and openly. They also point to the lack of available alternatives within organizations and nurses' strategic use of technologies to break through a culture of silence that puts patients, nurses and others at risk. Finally, our findings challenge the idea that external whistleblowing always comes at a cost to nurses. We found that the cost of remaining silent can be far greater, especially during a pandemic.

### 6.2 The role of whistleblowing tools

In light of the COVID‐19 pandemic, it becomes imperative to rethink our understanding of external whistleblowing and current approaches to whistleblowing. Traditionally seen as a process of disclosure initiated by an individual nurse, our findings reveal that this process can and should be facilitated by the development of whistleblowing tools such as the FIQ's online platform ‘*Je dénonce*’ [I denunce]. These tools can assist nurses in alerting the public, the media and government officials to wrongdoings while protecting their identity and preventing retaliation at an organizational level. As shown in our findings, such tools can also support collective solidarity and assist the work of nursing organizations. For example, nursing associations can amass evidence of policy failures to advocate more effectively and nursing unions can collect crucial evidence regarding workplace abuses even as they are locked out of their own bargained collective agreements. This being said, collecting information in itself is not sufficient. For these tools to be effective, they need to be part of a broader strategy towards greater transparency, accountability and responsiveness at the management, leadership and political levels. Without access to whistleblowing tools, nurses and other health care workers have relied heavily on social media platforms to blow the whistle. There is no denying that such platforms have played an important role during the COVID‐19 pandemic by supporting nurses' efforts to exchange information, signal wrongdoings, amplify whistleblowers' disclosures and support one another. However, their use raises a number of questions related to access, design, reach, impact and privacy that have yet to be studied in the context of whistleblowing in health care. In our case study, some nurses commented on the limits of social media platforms, including the risk of generating echo chambers that can, in turn, fuel exhaustion, hopelessness and a false sense of political efficacy.

### 6.3 The need for whistleblowing protection

In July 2020, Amon published a compelling commentary in The Lancet entitled ‘*Human rights protections are needed alongside PPE for health care workers responding to COVID‐19*.’ The commentary is consistent with our case study data. That is, there is a need to expand our thinking regarding the meaning of the term ‘protection’ to include layers of protection from COVID‐19 *and* means of protection from management, employers and governments who silence nurses and retaliate against those who blow the whistle. Protection against COVID‐19 is necessary, as our findings suggest, but they are not enough to ensure safe health care environments. It is worth noting that before COVID‐19, organizational cultures of silence, loss of accountability, lack of reciprocity and transparency in management structures and lack of protective policies and legislative structures had already created a context that is ripe for the occurrence of wrongdoing, poor responsiveness to reported concerns, as well as retaliatory practices against those who speak up (Perron et al., 2020b). Our findings suggest that these issues were significantly amplified during the pandemic and reinforce the need for enhanced protection.

Unlike other employees in the public sector, such as civil servants, Quebec nurses do not benefit from explicit whistleblowing statutory protections. Gruben and Bélanger‐Hardy (2020) pointed out that ‘whistleblower protection for health care workers [in Canada] continues to be piecemeal at best’ (p. 499). Their analysis of whistleblowing during the COVID‐19 pandemic supports the gaps identified in the case study data. First, at the regulatory level, explicit whistleblowing guidance wa lacking. Second, at an organizational level, the culture of silence across health care settings intensified during the pandemic. Third, at a statutory level, existing laws in Canada left nurses who blew the whistle unprotected.

Existing whistleblower protection laws still come up against the argument of duty of loyalty to the employer, which requires health care workers to approach whistleblowing from the perspective of the employee–employer relationship. While this duty of loyalty is prima facie compatible with duties arising from this relationship in the private sector, it is important to recognize that health care workers acting as public sector employees have additional duties to consider (Brunelle & Samson, 2005; Newham et al., 2021), especially in the context of a pandemic. Our findings show that the duty of loyalty of nurses is first and foremost directed towards the patients and the profession. As such, fulfilling their professional duties and protecting patients is more important than maintaining the reputation of their workplaces and employers. This, we argue, is an important part of the social contract between nurses and the public. Our position echoes the recent ruling of the Court of Appeal of Saskatchewan (2020) in *Strom v Saskatchewan Registered Nurses' Association* which reiterates the essence of this social contract and affirms nurses' right to speak out (and publicly):

Criticism of the healthcare system is manifestly in the public interest. Such criticism, even by those delivering those services, does not necessarily undermine public confidence in healthcare workers or the healthcare system. Indeed, it can enhance confidence by demonstrating that those with the greatest knowledge of this massive and opaque system, and who have the ability to effect change, are both prepared and permitted to speak and pursue positive change. In any event, the fact that public confidence in aspects of the healthcare system may suffer as a result of fair criticism can itself result in positive change. Such is the messy business of democracy. (para 160)

### 6.4 Strengths and limitations

This case study offers a significant contribution to the body of literature on whistleblowing in nursing, and it sheds light on important pandemic‐specific considerations that are relevant to decision‐makers, researchers and clinicians. The strengths of our study include the triangulation of three sources of data, the inclusion of a case description to situate the study findings and the selection of a unique case of nurses blowing the whistle with greater intensity than other Canadian provinces through different strategies and one novel, unique whistleblowing tool (the FIQ online platform). However, some limitations should be considered when interpreting our findings. The study was based in one province and may not reflect the reality of nurses in other jurisdictions. Sociodemographic information was only available for interview participants, which limited our understanding of the profile of nurses who blew the whistle. For example, most of our interview participants tended to be younger with less than ten years of nursing experience. We were not able to explore this further in the case study. Finally, the case study focused exclusively on the first wave of the COVID‐19 pandemic.

## 7 CONCLUSION

Over the course of the COVID‐19 pandemic, whistleblowing by nurses and other health care workers has intensified worldwide and has taken a turn outwards because of various governmental, organizational, managerial and technological factors (Amnesty International, 2020). Our case study offers a starting point to understand the experiences of nurses who blow the whistle during a pandemic. We have highlighted the importance of rethinking our understanding of external whistleblowing, developing tools to better support nurses and enacting legislated whistle‐blower protections that account for the nature of wrongdoings brought to the forefront during COVID‐19. Our findings reframe whistleblowing as a positive action rather than a negative one, one that nurses undertake as professionals committed to the public interest, as members of a collective and as workers endowed with basic, inalienable rights. They also further support a view of whistleblowing as a symptom of much broader problems of transparency and accountability. Addressing these problems is a crucial step towards protecting nurses and, therefore, the patients they care for.

## AUTHOR CONTRIBUTIONS

This case study was part of a larger study on whistleblowing for which the listed authors received funding (except CD). The case study design was led by MG. MG, AP, EM and CD collected and analysed the case study data: (1) News stories were collected by MG and analysed with AP, (2) Online forms were collected by EM and CD and analysed with MG and AP, (3) Interviews were conducted by MG and CD and analysed with AP. MG triangulated the data and identified the themes. MG wrote the original draft of the manuscript. All authors contributed to reviewing and editing the manuscript.

## FUNDING INFORMATION

This study was supported by the Social Sciences and Humanities Research Council, Insight Grant (435‐2019‐1249).

## CONFLICT OF INTEREST

We declare no competing interests.

### PEER REVIEW

The peer review history for this article is available at https://publons.com/publon/10.1111/jan.15365.

## Data Availability

News stories and online forms are publicly available. The interviews are not available for confidentiality reasons.
